# The Acquisition of Culturally Patterned Attention Styles Under Active Inference

**DOI:** 10.3389/fnbot.2021.729665

**Published:** 2021-10-05

**Authors:** Axel Constant, Alexander Daniel Dunsmoir Tschantz, Beren Millidge, Felipe Criado-Boado, Luis M Martinez, Johannes Müeller, Andy Clark

**Affiliations:** ^1^Theory and Method in Biosciences, University of Sydney, Sydney, NSW, Australia; ^2^Department of Informatics, The University of Sussex, Sussex, United Kingdom; ^3^Sackler Centre for Consciousness Science, University of Sussex, Brighton, United Kingdom; ^4^School of Informatics, University of Edinburgh, Edinburgh, United Kingdom; ^5^Institute of Heritage Sciences, Spanish National Research Council, Santiago de Compostela, Spain; ^6^Institute of Neurosciences, Spanish National Research Council, Universidad Miguel Hernández, Alicante, Spain; ^7^Institute of Prehistoric and Protoshistoric Archaeology, Kiel University, Kiel, Germany; ^8^Department of Philosophy, The University of Sussex, Sussex, United Kingdom; ^9^Department of Philosophy, Macquarie University, Sydney, NSW, Australia

**Keywords:** perception, culture, archaeology, active inference, eye tracking, simulation

## Abstract

This paper presents an active inference based simulation study of visual foraging. The goal of the simulation is to show the effect of the acquisition of culturally patterned attention styles on cognitive task performance, under active inference. We show how cultural artefacts like antique vase decorations drive cognitive functions such as perception, action and learning, as well as task performance in a simple visual discrimination task. We thus describe a new active inference based research pipeline that future work may employ to inquire on deep guiding principles determining the manner in which material culture drives human thought, by building and rebuilding our patterns of attention.

## Introduction

Do the worlds we build alter our own minds and the ways we process information? In one sense, it is obvious that they do—we read books, we listen to our teachers, and learn new ways of thinking and reasoning as a result. Thanks to lifelong learning, we may become experts in a domain such as forestry and become able to attend to, and differentiate, new things as a result. But we are also immersed in a sea of material structures and artefacts such as pottery, ceramics, clothing, buildings, tools, and more. As we encounter and explore these artefacts and structures, they too influence our patterns of visual and embodied exploration, and thus our learning. But the nature and potential cognitive importance of these interactions with material structure remains ill-understood.

Iterated encounters with non-linguistic aspects of material culture, we believe, do not simply reflect human thinking and reasoning—rather, they shape and alter it. Our minds are as much the products of these materialities as the cause. This is a bold claim, yet one that is quite often found in the sciences of mind and culture (Dennett, 1991, 1996; Clark, 1997; Sutton, 2002; Knappett, 2005; Renfrew and Malafouris, 2010). To our knowledge, it is a claim that has not been experimentally demonstrated or subjected to rigorous analysis and testing. The simulations we report below are meant as a first step towards building a pipeline to explore and test this claim—that encounters with non-linguistic artefacts can alter patterns of thought and attention in cognitively interesting and beneficial ways.

With this goal in mind, our paper presents a proof of principle for modelling visual foraging and sensory learning of artefacts using active inference for Markovian inference models. Markovian models are used to perform predictive statistical inference over some states of interest, given the outcomes those states are known to generate. For instance, Markovian model can be used to perform weather forecasting over some unknown states (a.k.a. hidden states, or unknown variables) which would represent the weather for each day of the week (e.g., rainy; sunny), and where the outcomes would be some observable property of the possible states (e.g., cloud shapes). After observing these properties, inference proceeds by combining known prior probabilities of transitioning from one state to the next (e.g., history of transitions between rainy to sunny days, or sunny days to sunny days, etc.) with the known likelihood of observables under each state. The resulting posterior specifies the Bayesian probability the hidden variables at the next time step (e.g., tomorrow's weather). In this paper, we utilise the theory of active inference to perform the requisite inference, action-selection, and learning for our model.

Crucially, active inference straddles multiple levels of Marr's hierarchy, from deep computational considerations in statistical inference and thermodynamics, all the way down to being able to build biologically plausible models of psychophysical tasks which accord with known neurophysiology. This theoretical breadth licences us to create an active inference model of the interaction between inference and learning systems and material culture which can demonstrate, through psychophysical observables, the importance of the material world for shaping mental attention styles and ultimately cognitive capacities.

We present two experiments. In experiment 1, we provide a pilot simulation of visual foraging that showcases the potential of our method for modelling empirical data on foraging over differentially complex cultural artefacts. Experiment 1 can be viewed as a training experiment, where artificial agents learn about the hierarchical structure of artefacts, and where this acquired knowledge is later applied to a categorisation task (experiment 2). Our simulation focuses on implementing artificial behaviour that could mimic *in vivo* participants scan paths over antique vases such as observed in Criado-Boado et al. (2019). Criado-Boado and colleagues studied the influence of decoration patterns on scan paths employed by visual foragers. They use a vertical index (Vi) to measure the influence of various patterns on visual saccades, relative to the size of the visual display presenting the differentially decorated vases (e.g., more or less complex decoration painted horizontally or vertically).

We show that an increase in decoration complexity, when modelled as patterns of hidden states, entails characteristically different scan paths, and hence Vi; We call these scanpaths “Culturally Patterned Attention STyles” (C-PAST). These scan paths are the result of the agent attempting to predict the next decoration based on observed pigments and learning the probability transitions between the visual motifs forming the decorations. These scanpaths should be viewed as heuristics of culturally shaped patterns of attention. Future work should attempt to fit the model generating those scanpaths with real participant data. The motivation for calling the scanpaths “cultural” is that vase decorations have been shown to be a good indicator of cultural complexity. Hence, we call “cultural,” or rather “culturally patterned” the scanpaths that result from learning based on the exposure to such decorations; the patterning here being synonym of learning.

In experiment 2, we provide a modelling method to transfer the learning of priors across simulations under active inference, which, to our knowledge, has never been done before in the literature. Transfer learning here refers simply to the transfer of knowledge across tasks (e.g., employing knowledge acquired in task X to perform the actions required in task Y). The challenge with the transfer of learning in active inference modelling is that the model parameters (e.g., transition probabilities between hidden states) are normally task specific, which means that they correspond to the environment of the task at hand (e.g., motifs as hidden states being specific to the vase perceived by the agent). The novel modelling strategy we propose in this paper allows transfer learning by breaking down the environment of a task into units that are general purpose hidden states. These units are locations in a discrete 2-dimensional map, which we call the remapping likelihood matrix (see method for details). The remapping matrix allows us to local representations of the immediate environment, and, crucially, to reuse these units or groups of units, when learned, across tasks. While it unlocks the possibility to accomplish our simulation, we recognise that the present method of likelihood remapping is trivial. Based on the learning of the structure of vases' decorations in experiment 1, in experiment 2 we simulate a pattern categorisation task that involves reusing learned model parameters in experiment 1. In the categorisation task, the agent has to match a series of motif cut-outs with their corresponding motif. We show how performance (hits vs. non-hits) changes depending on learned parameters under the different levels of cultural complexity afforded by vases transferred from experiment 1.

In summary, with experiment 1 and 2, we show the potential of active inference to study (i) exposure to artefactual complexity leading to the acquisition of the knowledge underwriting different Culturally Patterned Attention STyles (C-PAST)—here knowledge about transition probabilities among hidden states, or representations of the structure of the world; (ii) the repurposing of C-PAST in novel cognitive tasks, and the manner in which different C-PAST influence performance in novel cognitive tasks. We are aware that the task that we use may be considered too simple to demonstrate the effect of C-PAST on cognitive task performance, and that our task is limited to non-natural scenes. However, the goal of our simulation, beyond reproducing the results of Criado-Boado et al. (2019) is to provide a simple example of a scalable modelling strategy for future research on related issues in the field of cognitive archaeology.

## Vertical Index, Social Complexity, Cultural Complexity and Attention

The vertical index (Vi) is a measure that compares the proportion of horizontal to vertical saccades made when viewing an image (Criado-Boado et al., 2019; Millán-Pascual et al., 2021). This measure, which is closely related to the density of information presented in vertical dimensions, has been shown to vary considerably across items ranging from pots to monuments, drawn from different archaeological epochs (Prieto-Martínez et al., 2003). The archaeological record shows that decoration patterns of complex prehistoric societies generally followed high Vi patterns, whereas low Vi patterns are found in simpler societies. Criado-Boado et al. (2019) note that archaeologists accept that the evolution of pottery decorations parallels, in the particular chronological sequence on study, changes in the level of complexity of social organisation [see Prieto-Martínez et al. (2003) for a detailed characterisation of the social complexity embedded in the pottery sequence analysed in Criado-Boado et al. (2019) and see Criado-Boado (2014)] for a more general and theoretical account of the interactions between materiality and social processes] and propose that a virtual index of decoration may be a measure or reflection of such a social complexity [Müller et al. (2015) also illustrates similar conclusions for a different pottery style]. Criado-Boado et al. (2019) found that the verticality of decoration correlated with the chronological evolution of the decorations on ceramics displayed in their study; these being associated with difference cultural periods and associated levels of social complexity. They showed that eye movements of participants followed the same evolutionary trend reported by the Vi index when presented with the vases' decorations characteristic of each successive social periods.

Here, what we refer to as social complexity differs from what is sometimes described as cultural complexity (Sterelny, 2020). Social complexity denotes the overall level of organisation of a society, whereas cultural complexity denotes the complexity of artefacts found in a given population. Cultural complexity is sometimes viewed as a proxy to social complexity, as it would reflect the level of skills and expertise of the tool and artefacts makers and users, which in turn would reflect the level of social complexity. Cultural complexity can be viewed as either repertoire complexity, or peak complexity (Sterelny, 2020). Repertoire complexity corresponds to the number of distinct tools that were used in each society, whereas peak complexity corresponds to the level of complexity of a given tool, which can be measured in terms of parts and functions of the tool; these being called technounits Oswalt (1973). The correlation observed by Criado-Boado et al. (2019) was between social complexity and the Vi of decorations on vases. The correlation was not between social complexity and cultural complexity.

A challenge with studying the relation between social complexity and cultural complexity is that repertoire complexity and peak complexity may vary independently (Sterelny, 2020), and depending on the sort of artefact one considers, peak complexity may even be inversely proportional to the true level of skills of artefacts makers reporting social complexity. Moreover, the locus of peak complexity may change over time in a same society. These problems are especially salient when considering the complexity of aesthetic objects like vase patterns. For instance, it is common to observe disparity within the artefactual repertoire, with simpler societies having poorer decorative vase patterns but highly complex body ornamentations like tattoo motifs or plumes arrangements. The same applies to more advanced societies and pottery decorations, whose peak complexity can correlate at first with the level of social complexity, but then decrease with time as the society discovers new material and media for artistic expression (e.g., jewellery, metallurgy, architecture, etc.). For instance, pottery was important to express social styles and social identities in the Atlantic façade between 6,000 and 2,000 BP, while in other cultures and times other sort of material were used to mainly express social identity (e.g., jewellery, metallurgy, monuments, or tattoos, personal ornaments, or plumes).

Despite the intrinsic interest of these issues and their importance for understanding the historical record, it is important to note that our target in the simulation studies is something rather different. Our goal is to explore the potential role of cognition (attention, perception and learning) as a variable operating within these complex regimes. Specifically, we are asking whether, and in what ways, interactions with artefacts might alter patterns of attending, which in turn alter ways of thinking and reasoning about the world (e.g., in a cognitive task). Thus, we introduce cognition (attention, perception and learning) as a third variable to the complex relation between social complexity and cultural complexity. The hope is that styles of cognition may function as an explanatory bridge between cultural and social complexity. Accordingly, our simulation explores the synthetic relationship between task performance and the acquisition or learning of attention styles based on the exposure to vase decorations. The motivation for this simulation is to explore the ways interactions with artefacts might alter patterns of attending, which in turn alter ways of thinking and reasoning about the world (e.g., in a cognitive task). If such effects are real, then there may be a good reason to believe that there exists a link between the structure of the human-made world and the ways we think and reason after cultural immersion in different such worlds. Because artefacts affording greater vertical index correlate with social complexity, and because vertical indices illicit characteristically different visual foraging patterns (Criado-Boado et al., 2019), one could hypothesise that there is a ratchetting loop between the acquisition of attention styles, features of the artefacts that illicit such an acquisition (e.g., Vi), and cultural complexity.

Note that novel patterns of attending do not necessarily witness of a neurobiological change in the human evolutionary history (e.g., encephalization). The hypothesis on the cognition-culture loop is not primarily a gene-culture co-evolutionary hypothesis on the evolution of social complexity and cultural complexity (e.g., Henrich, 2015). Rather, such a hypothesis refers to dynamics at the level of cognition and culture. Attention styles are acquired over developments; they are akin to cognitive “gadgets” (Heyes and Frith, 2014) that support the scaf-folding of more complex abilities such as language and mind reading. One of these abilities may be that of reproducing complex human social ensembles; an ability scaf-folded through artefactually mediated acquisition of attention styles. The current simulation is a first step towards studying such cognition-culture loop under the theory of active inference.

Finally, note that our project differs from related research in the field of active inference, culture and cognition. Here, our goal is not to account for the formation and function of human culture, but rather, to inquire on the manner in which culture shapes perception and influences task performance. That is, we are not here attempting to define what culture is and how it works, but rather we are here attempting to describe the way humans may respond to its products and how those influence cognitive task performance. While this latter problem is certainly part of the more general project of accounting for the formation and function of culture, this problem remains one that can be approached independently of the larger discussion on the ontology of culture. Under active inference, the ontology of culture is defined as patterns of attention, or “regimes” of attention shaped by local practises (e.g., Kaufmann and Clément, 2007; Ramstead et al., 2016; Constant et al., 2019, 2020; Veissière et al., 2020). Despite the differences in research orientations noted above, our simulation may be viewed as providing one possible illustration of the manner in which the acquisition of regimes of attention (here C-PAST) influences task performance.

## Method: Active Inference

Active inference is a theory arising from theoretical neuroscience, which posits that perception, action, and learning can be fundamentally united since they can be cast as performing a form of approximate Bayesian inference (known as variational inference) on the same information—theoretic objective (Friston, 2010). Although anchored in abstract conceptions of inference, active inference possesses a neurobiologically plausible process theory (Friston et al., 2017), and has been applied to explaining and building models of diverse aspects of neural and cognitive function such as planning and navigation under uncertainty (Kaplan and Friston, 2018), saccade generation and reading (Parr and Friston, 2017), sequential decision making tasks (Friston et al., 2013, 2016), up to complex continuous control tasks (Pio-Lopez et al., 2016; Fountas et al., 2020; Millidge, 2020; Tschantz et al., 2020), as well as psychophysical observables such as modelling evidence accumulation (FitzGerald et al., 2015). Moreover, through the expected free energy functional, active inference also entails a natural epistemic drive which has been exploited before in previous active-inference studies of visual foraging (Friston et al., 2015; Mirza et al., 2016). Here, we present a high-level description of active inference. For a detailed overview of active inference in discrete state-spaces and for the purpose of economy of space, we refer the technically minded reader to the dedicated method papers of Friston et al. (2015, 2017), and Da Costa et al. (2020).

### An Overview of Variational Inference

Active inference posits that action, learning and perception can all be described as a process of variational inference. Variational inference is an approximation to exact Bayesian inference which postulates the existence of a variational recognition density, which is matched to the true posterior via an optimisation process. Variational inference thus converts a difficult and intractable inference procedure into a potentially tractable optimisation process, for which good approximate solutions exist. Variational inference obtains its solution by minimising the variational free energy functional, and this is used in our model for perception—i.e., the inference of hidden states from observed outcomes. Active inference extends this theory to include action, which is inferred from preferences over sequences of potential future states. This requires the use of a subtly different objective functional—the expected free energy—which is a functional over expected future states and observations. The expected free energy naturally includes an epistemic exploration-inducing information gain term which encourages active inference agent's to seek out novel outcomes, which thus can mimic key behaviours in visual foraging which is all about information gathering.

Variational inference depends on two mathematical objects—the variational recognition distribution (hereafter referred to as the variational distribution) and the generative model. The variational distribution is a distribution over all hidden variables in the model and represents the agent's beliefs about the state of the world. The generative model is the agent's model of how the observables in the world are “generated” by the hidden variables which must be inferred. During inference, the variational distribution (the agent's beliefs) are optimised to best conform to the outcomes or data observable by the agent. Thus, in inference, the generative model is “inverted” —in that we look to recover the mapping from observations to hidden states, given a mapping from hidden states to observations.

### An Overview of the Variational Distribution

Formally, let x refer to hidden variables, where *x*^n^ refers to the hidden state at level n, and π refer to a policy (fixed sequence of actions). The variational distribution can then be factorised as follows:


(1) $$\begin{array}{l} Q\left(x_{0:T}^{1},x_{0:T}^{2},\pi \right)=Q\left(x_{0}^{1}\right)Q\left(x_{0}^{2}\right)Q\left(\pi \right)\overset{T}{\underset{t=1}{\prod }}Q\left(x_{t}^{1}|\pi \right)Q\left(x_{t}^{2}|\pi \right) \end{array}$$

Moreover, the agent's generative model can be factorised as:


(2) $$\begin{array}{l} p\left(o_{0:T},x_{0:T}^{1},x_{0:T}^{2},\pi \right)=p\left(\pi \right)p\left(x_{0}^{1}\right)p\left(x_{0}^{2}\right)p\left(o_{0}\right)\\ \overset{T}{\underset{t=1}{\prod }}p\left(o_{t}|x_{t}^{1}\right)p\left(x_{t}^{1}|x_{t-1}^{1},x_{t}^{2},\pi \right)p\left(x_{t}^{2}|x_{t-1}^{2},\pi \right) \end{array}$$

Given these distributions, inference is achieved by optimising the variational distribution in order to minimise free energy:


(3) $$\begin{array}{l} \begin{array}{c} Q*\left(x_{0:T}^{1},x_{0:T}^{2}\right)=\text{argmin}F\left(Q_{0:T},o_{0:T}\right)\\ F=D_{KL}\left(Q\left(x_{0:T}^{1},x_{0:T}^{2};\phi \right)||p\left(o_{0:T},x_{0:T}^{1},x_{0:T}^{2}\right)\right) \end{array} \end{array}$$

In a similar fashion, action selection is achieved by optimising the variational distribution to minimise expected free energy, which we compute at each step:


$$\begin{array}{c} \pi \sim Q^{*}(\pi )=\arg\min_{\pi }\varsigma (Q,\pi )\\ \varsigma (Q,\pi )=E_{Q(o_{t:T},x_{t:T}^{1},x_{t:T}^{2}|\pi )}[D_{KL}Q(x_{t:T}^{1},x_{t:T}^{2}|\pi )\\ \left||p(o_{t:T},x_{t:T}^{1},x_{t:T}^{2}\right)] \end{array}$$

### An Overview of the Generative Model

In the current work, the active inference agent utilises a two-level hierarchical generative model parametrised by four matrices “A^1^,” “A^2^” and “B^1″^ and “B^2″^ (for a deeper description of hierarchical models in active inference see, Friston et al., 2017). Here we present the role these matrices play in the variational inference over states, future states and outcomes in general. In the result section, we describe the semantic of these matrices, which will be specific to the tasks we seek to accomplish in experiments 1 and 2.

The “A” matrices represent the parameters of a likelihood distribution which maps from the hidden states at a hierarchical layer to the outcomes associated with the layer (the outcomes of all hierarchical layers other than the lowest layer correspond to the hidden states of the hierarchical layer below). These matrices denote the instantaneous probabilistic mappings between the hidden states and outcomes.

The “B” matrices represent the (policy- dependent) transitions between the hidden states over multiple time-steps. The parameters of the “B” matrices were learnt through experience. This learning can be cast as inference on the parameters of a dirichlet hyperparameter over the entries of the “B” matrix. For more details see Da Costa et al. (2020). Crucially, the parameters of the “B^2″^ matrix are inferred over the course of a trial and provide the representation of the C-PAST.

Given such a generative model and an initial state distribution, sequences of potential future outcomes and hidden states can be generated and compared for different potential policies (sequences of action) which could be enacted. These sequences of future outcomes and hidden states are scored by the expected free energy functional (denoted “G”). Policies are selected which minimise “G.”. In our experiments, look-ahead was only performed for a single time step into the future and actions were selected which greedily minimised “G.”

Another important aspect is the encoding of an agent's preferences into the generative model. This is encoded through the matrix “C” which specifies a desired probability distribution over outcomes. In our experiments the agent strongly desired to observe pigments and will be averse to observing non-pigments. This simple constraint on agent behaviour is sufficient to generate complex visual-foraging behaviour.

**Figure 1:**
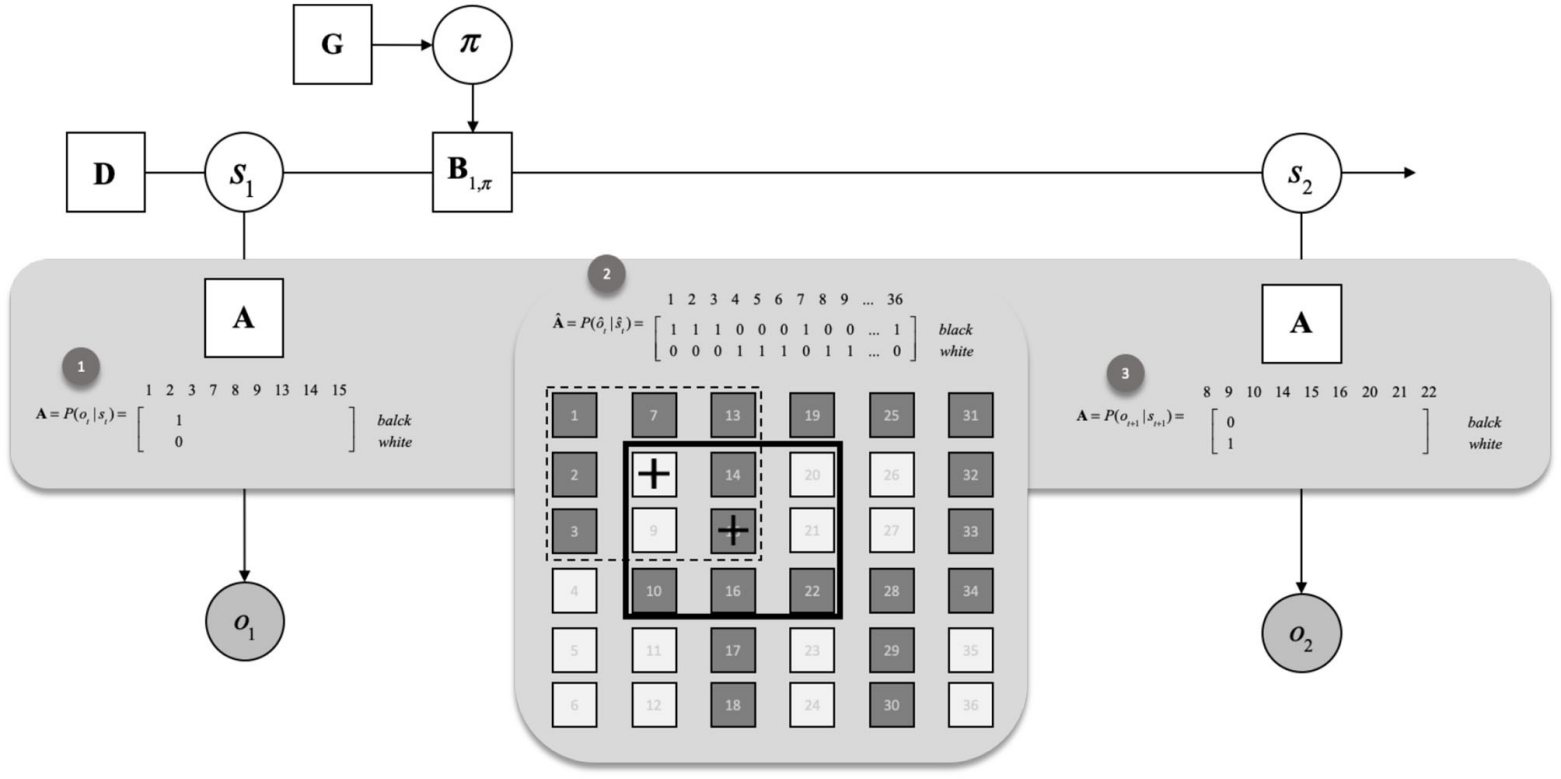
Likelihood remapping. For illustrative purposes, this figure presents a generative process (A hat) made of 36 locations, or states. Each state is associated with an outcome, either black or white. (1) the initial likelihood is defined for an agent that would start in location 8. The likelihood of the generative model is specified based on the 8 locations, or hidden states surrounding the current location, as well as the current location. (2) Based on the inferred policy (e.g., 8 → 15), we move the agent in the generative process, here, to location 15. (3) Indexing the novel surrounding and current location from the generative process, we remap the likelihood that will be used at t + 1 to infer the state and the policy.

We specify a prior the entries of the “A” matrices. Crucially, to ensure that the active inference agent only was in possession of local knowledge (i.e., the content of its foveated region and not the entire image), we utilised the novel likelihood remapping trick by which the “A” matrices were represented in a state-dependent fashion so that the agent was only aware for a given hidden state (location), the presence or absence of the pigment in a 3 x 3 square around the agents location. Likelihood remapping allows the agent to perform state inference and navigation by bypassing the full representation of the generative process (i.e., environment). This is in contrast to standard active inference approaches which typically require the agent to be given a correct global understanding of the scene. To achieve this locality, the “A” matrix becomes state-dependent so that it only provides information about outcomes in the proximity of the state the agent is in. A further description of this likelihood remapping method can be seen below in Figure 1.

## Results

### Experiment 1

**Figure 2:**
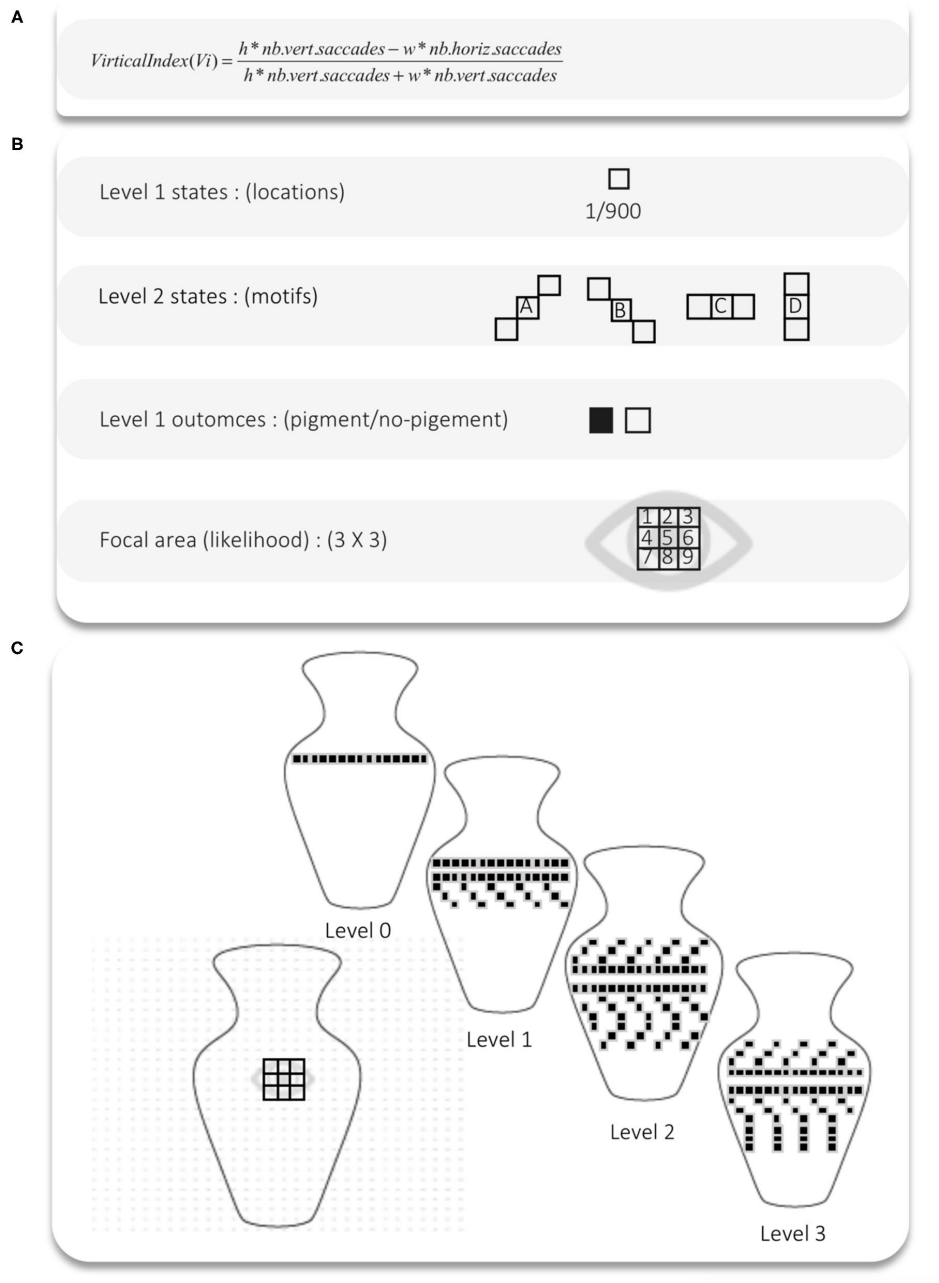
**(A)** Vi is a measure of visual saccades relative to the size of the visual area gazed upon. This area increases with decoration complexity. **(B)** States and outcomes for the generative model. At level 1, the outcomes are the absence or presence of a pigment. The states are the locations (1/900) of which we take a sample of nine currently available locations to define the focal area (updated based on the navigation matrix for every trial, see method). Level 2 states are motifs (sets of locations) and their associated outcomes are the location at level 1 [e.g., “P(location|motif)]. The focal area corresponds to the outcome likelihood at level 1. The important thing to remember is that we are respecifying the likelihood after each eye movement, using the remapping likelihood matrix (see method section). **(C)** 900 locations grid over which the active inference agent scans. The agent can decide to move from the central location of the 3 x 3 focal area (grid) to any location of the grid. The four levels of decoration complexity build on one another. Level 0 is a straight line, and level 1 adds verticality by adding oblique shapes below the line. Level 2 adds oblic motifs on the top of the straight line as well, and level 4 adds vertical lines below the oblique shapes at the bottom.

The goal of experiment 1 was to exemplify the relation between artefactual complexity and scan path cultural specificity under active inference. We showed how variations in artefactual complexity leads to the acquisition of different “Culturally Patterned Attention STyles” (C-PAST). Scan paths are artificial visual saccades enacted by the agent during the visual foraging task. The goal of the visual foraging task was simply for the agent to explore the visual scene, which consists of a vase decorated with motifs made of pigments. The agent's simulated gaze starts at the centre of the vase and is free to explore the vase for 100 timesteps. We presented the simulated agent with vases that had different levels of complexity—that is, that were made up of more or less visually rich patterns. The richness of the patterns came from the inclusion of more or less vertical features, or motifs, from horizontal (0 degree angle), to oblique (−45 and +45 degree angle), to vertical (90 degree angle). We measured the influence of pattern complexity on visual foraging with a version of the virtual index (Vi) used in Criado-Boado et al. (2019). Vi is a measure of visual saccades relative to the size of the display upon which the vase is presented. The empirical results of Criado-Boado et al. (2019) suggest that vase complexity affects change in scan paths' Vi. The purpose of simulation 1 was to reproduce this effect in silico and based on the parameters needed to simulate the effect, phenotype the different attention style or C-PAST acquired through exposure to vases with four levels of complexity (0 to 3) (see Figure 2).

#### The Model for Experiment 1

To perform the task in experiment 1, the simulated agent applies our inference algorithm to a simple two level Markovian generative model. The generative model allows the agent to infer two things: (i) the hidden states at level 1 or 2, and (ii) an action policy, which optimises the desired sequence of hidden states enacted by the agent. In experiment 1, the level 1 hidden states are locations on the visual display where pigments can be found. The presence or absence of a pigment functions as the sensory outcome. Level 2 hidden states represent the motifs which consist of repeating patterns of pigments, for instance, crosses, diagonal and horizontal lines (see Figure 2). Using a two-level hierarchical generative model allows us to simulate an agent that can infer the presence of more abstract hidden states (i.e., level 2 motifs) based on its inference of simpler hidden states (i.e., level 1 pigments). For each cycle of inference at level 2, four cycles of inference are performed at level 1, that is, four pigments are inferred. The heatmap we present below is the result of having inferred those different hidden states. The second thing the agent can infer based on its generative model is an action policy, which here stands for a (sequence of) visual saccades. Action policies are simply sequences of control states that are inferred over multiple time steps based on preferences the agent has for certain outcomes.

The generative model represents and performs inference over four sets of parameters. The first is a likelihood parameter A^1^, which exists at level 1, and is a probabilistic mapping between sensory outcomes (pigments) and level 1 hidden states (locations on the visual display). At level 1, we keep the likelihood deterministic (all [0 1]), which speaks to the fact that the agent can clearly perceive the pigments. The second likelihood parameter A^2^, represents the probabilistic mapping between the motifs and the locations perceivable by the agent. This likelihood is also deterministic, which speaks to the fact that the agent knows how a given motif (for instance a cross) can be represented by a sequence of pigments. The second set of parameters are the transition probability mappings between level 1 hidden states B^1^ or the level 2 hidden states B^2^. In experiment 1, the agent learns the transitions between hidden states (motifs) at level 2. Transitions are deterministic at level 1 and depend entirely on the action policy. A two-level model, with uncertainty in level 2 transitions (motifs transitions) will scan differently. Increases in vase complexity drive the learning of motifs transition (i.e., patterns). We refer the reader to the method section for the details of the manner in which our inference algorithm performs the inferences, formulates action policies, and learns B^2^.

#### Vertical Index

The Vertical index (Vi) is defined as the height “h” of the region upon which the agent gazed times the number of vertical saccades (number of steps taken vertically given the inferred policies), minus the width “w” of the region gazed upon time the number of horizontal saccades, all that divided by the sum of the product of the height “h” and number of vertical saccades, and the product of the width “w” and the number of vertical saccades:


(4) $$\begin{array}{l} VirticalIndex\left(Vi\right)=\frac{h*nb.vert.saccades-w*nb.horiz.saccades}{h*nb.vert.saccades+w*nb.vert.saccades} \end{array}$$

**Figure 3:**
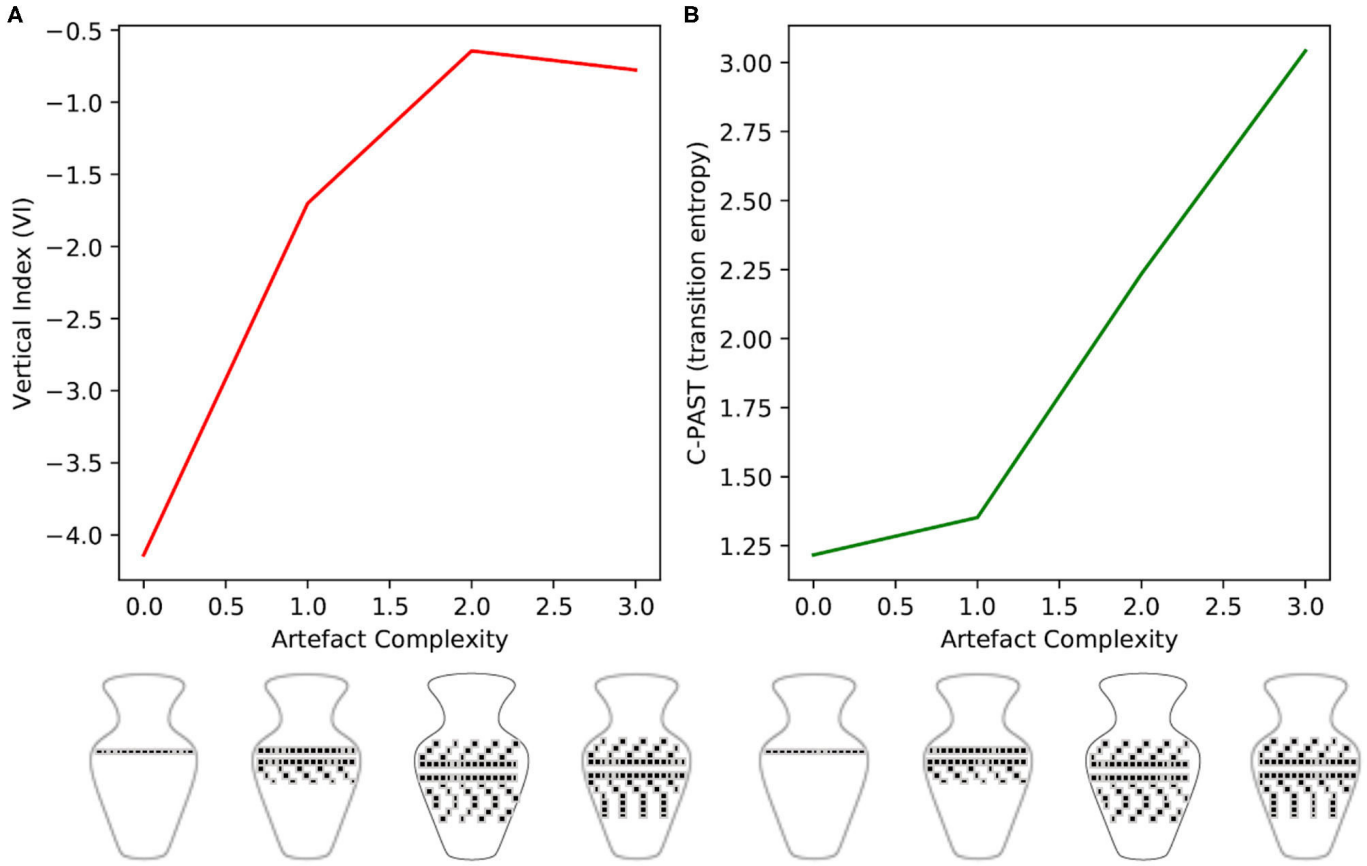
**(A)** The effect of artefact complexity on the vertical index (Vi) measure of scan paths. Consistent with the empirical findings of Criado-Boado et al. (2019), we find that artefactual complexity positively correlates with Vi **(B)**. The effect of artefact complexity on the C-PAST measure of learned generative models. As described in the main text, this measure quantifies the entropy of the motif-transition parameters “B2,” which are learned over the course of the 100 trials. These results demonstrate that artefact complexity correlates positively with C-PAST, highlighting the symmetry between environmental complexity and model complexity.

**Figure 4:**
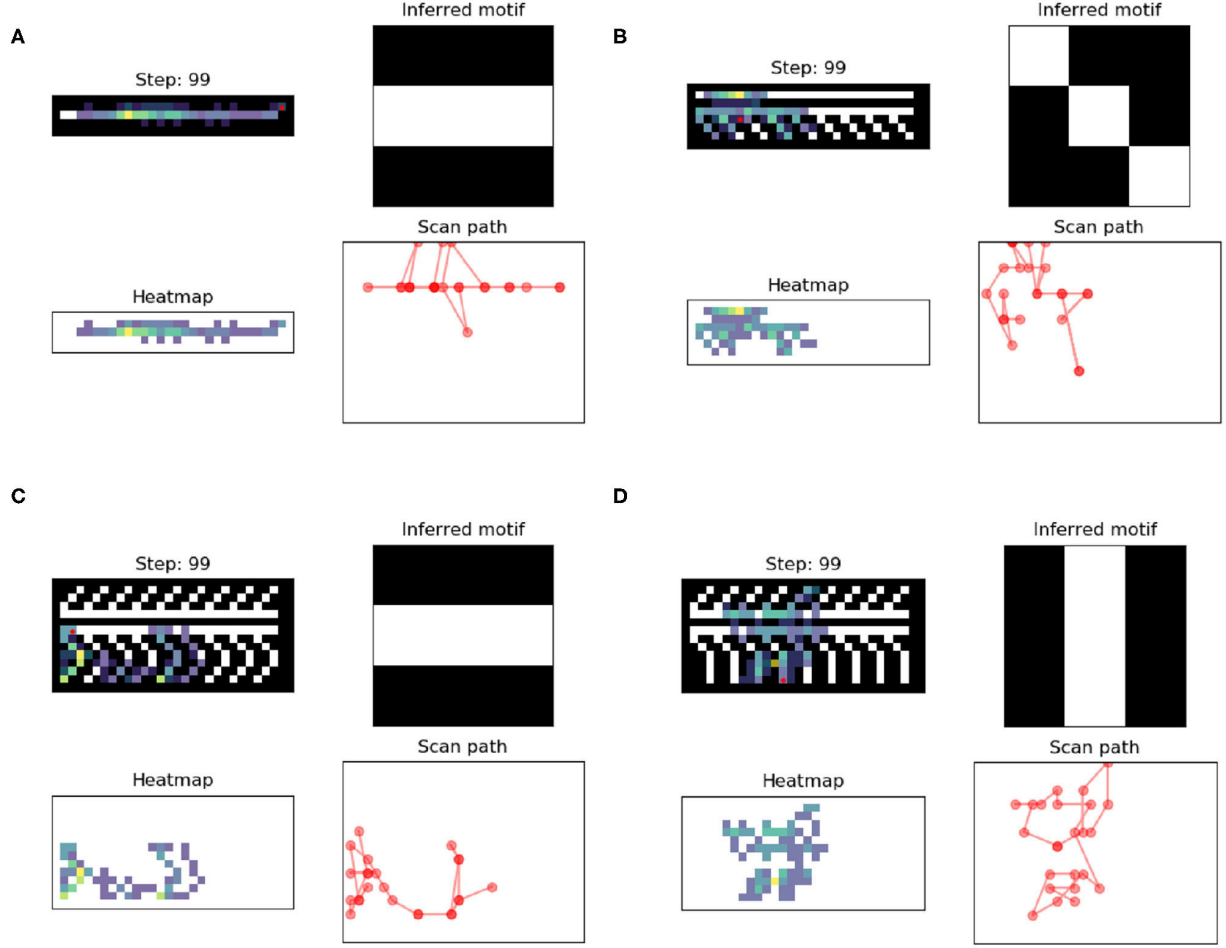
For each panel [**(A–D)**, or complexity 0 to 3], the upper left quadrant represents the heatmap and the final location (red dot), with the motifs superimposed at the end of the 99 trials; the lower left quadrant represents the heatmap alone at the end of the 99 trials; and the lower right quadrant represents the scan path over 99 trials; the upper right quadrant represents the final motif perceived. The heatmap represents the paths and the number of times the agent moved over a location. The more often a location was gazed upon, the yellower it became (gradient from blue to yellow). MP4 versions of the experiment can be found at https://github.com/BerenMillidge/MaterialCulture.

The change in Vi relative to the four levels of complexity are presented in Figure 3, with their associated level of decoration complexity. GIF representations of the simulation as well as the source code for all experiments can be found at https://github.com/BerenMillidge/MaterialCulture. The results show that Vi correlates positively with the levels of complexity, as expected, and empirically observed in Criado-Boado et al. (2019). We present the scanpaths in Figure 4.

#### C-PAST

We define the “Culturally Patterned Attention STyles” (C-PAST) as sets of motif-transition parameters learned when the agent is presented with decorations during exploration. Our simulation shows that difference in pattern complexity naturally entails differences in C-PAST, leading to systematic differences in Vi (Figure 3). To measure the C-PASTs, we use the entropy, in information theoretic terms, of the sets of motif-transition parameters B2. Formally, we define our measure of C-PAST as


(5) $$\begin{array}{l} C-PAST=H\left[B^{2}\right]=-\overset{N}{\underset{i}{\sum }}B_{:i}^{2}\log B_{:i}^{2} \end{array}$$

where H is the Shannon entropy and N is the number of motifs. We use entropy because it allows us to describe intrinsic features of the distributions, without having to commit to a normative assessment of those distributions (e.g., compared to an ideal, extrinsic criterion of goodness). Indeed, the purpose of measuring C-PASTs is simply to phenotype the various attention styles that obtain from the exposure to various levels of cultural complexity. Note that we only allowed for learning of transition probabilities (B2 parameters), but in principle, nothing prevents one from allowing learning in other parameters so as to get a richer measure of C-PAST (e.g., entropy of A and B parameters).

### Results Experiment 2

**Figure 5:**
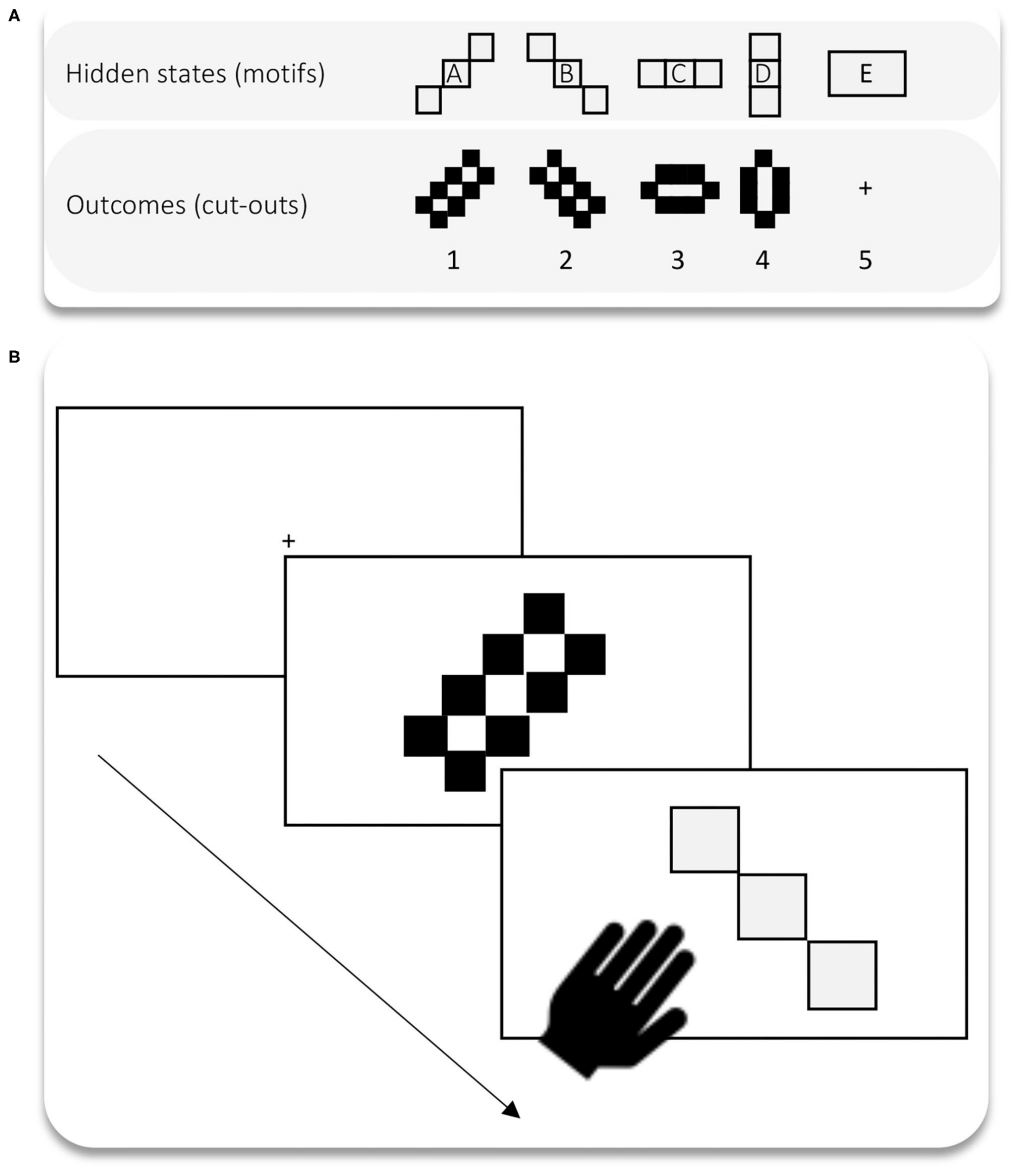
**(A)** States and outcomes are the shapes and the cut-outs respectively. There are no preferences for specific cut-outs; only aversion for the outcome that corresponds to the “+”. This is to make sure that the simulated agent always makes a decision across the 100 trials. **(B)** The task wherein the agent is presented with a series of cut-outs (outcomes) and has to infer what shape should fit in. We ran 100 trials where the agent is presented with a blank display followed by a cut-out. A single trial has three moments: (i) the agent is presented with a target (first slide); (ii) the agent receives the sensory entry, or cue that corresponds to the cut-out (second slide); (iii) the agent infers and selects the motif that matches the cut-out (slide 3).

The goal of experiment 2 was to explore the impact of different C-PASTs on cognitive task performance in a novel cognitive task. Note that the learning only happens in experiment 1. This means that we simply import the trained or learned parameters into the experiment two without letting the model further learn within the context of experiment 2. Accordingly, experiment 2 is not a typical transfer learning experiment. However, the proposed setup is ready for bone fide transfer learning simulations as future work could allow for learning, and thus study the effect of transferred learning on learning and task performance. Here, we only focused on the effect of prior learning on novel task performance. Experiment 1, which could be viewed as a “training,” or learning experiment was a visual foraging task. Experiment 2 is a simple visual classification task where the agent is presented with a predetermined series of cut-outs of certain shapes and must select the shape that matches the cut-out (see Figure 5). We simulated the task under the four different C- PASTs acquired in experiment 1. These were acquired through the exposure to the four different levels of decoration complexity on the vase. We presented the same predetermined series of cut-outs to all agents. We then recorded hits and non-hits over 100 trials, or series of 100 cut-outs. The agent received no feedback on its answer, meaning that no further learning took place in experiment 2.

#### The Model for Experiment 2

We use the same model as in experiment 1, but with a single level of parametrisation. Hidden states correspond to the shapes that made up the motifs in experiment 1, and the sensory outcomes are the cut-outs. Accordingly, the transition matrices (B parameters) are the transition probability mappings between shapes, and the likelihood parameter (A parameter) is a likelihood mapping between cut-outs and shapes. The likelihood mapping is deterministic, meaning that our agent can perfectly sense the shapes and their associated cut-outs (i.e., the agent has a non-noisy sensory access to cut-outs). The mappings for the transition parameters are those that have been learned in experiment 1 (i.e., as level 2 transition probability mappings, or level 2 B parameters B2), and so may contain uncertainty. Crucially, and distinct in this work is that we use a novel method of “likelihood remapping” to ensure that the agent at any point only perceives its local environment—i.e., the central foveated region of the visual.

#### Performance

**Figure 6:**
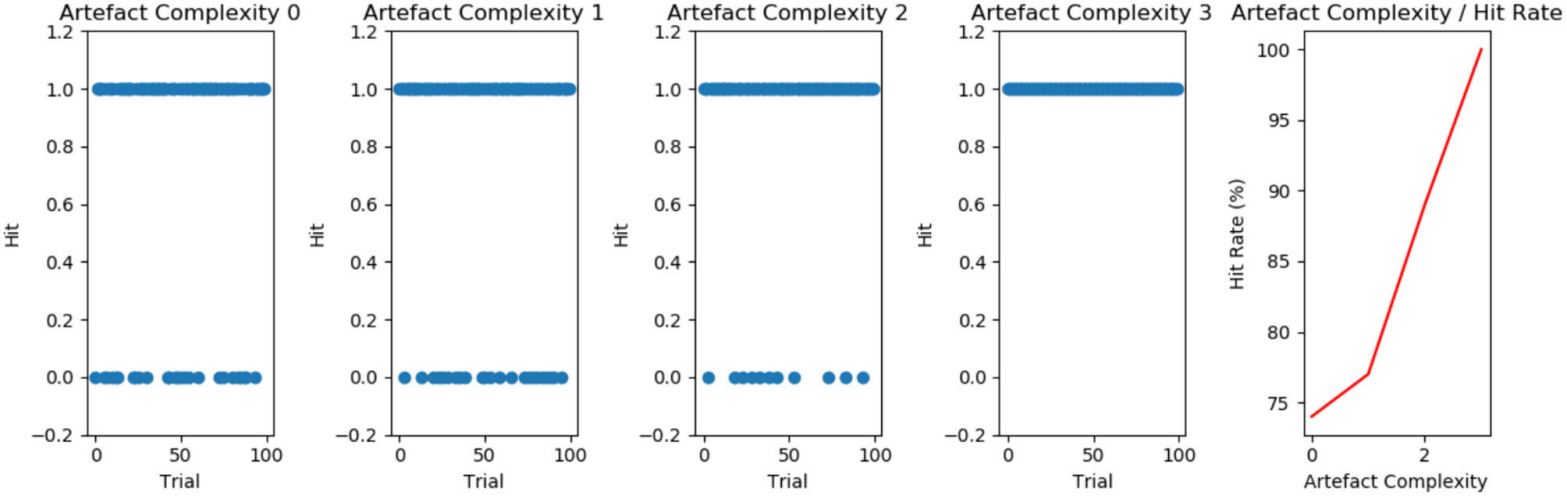
From left to right: Hits (1.0) vs. non-hits (0.0) over 100 trials for agent's exposed to different levels of artefact complexity. We find that the accuracy of agent's guesses (percentage of hit-rate) correlates with the level of artefact complexity.

The stimuli we employed in experiment 2 was a series of cut-outs. The task was to select the matching cut-out. We recorded the number of hits and non-hits over 100 trials. Figure 6 presents the results for the agents having been trained under the four different levels of decoration complexity in experiment 1. Our results show that C-PAST trained under higher levels of cultural complexity leads to increased performance.

## Discussion

This paper presents two computational experiments using active inference. The first was a training simulation wherein an agent could freely explore the decorations on four different vases affording four different levels of decoration complexity. Decoration complexity was defined in terms of the amount of verticality in motifs as well as the number of motifs. We used the decoration complexity to train different culturally patterned attention styles (C-PASTs), which we phenotyped in terms of the entropy of the distribution of their associated parameters. We observed that the different C-PASTs correlated with different virtual indices (Vi). The overall observation was that the increase in complexity correlated with increase in Vi, as observed empirically by Criado-Boado et al. (2019). Complexity and Vi also correlated positively with C-PASTs' entropy. The goal of our simulation study was simply to reproduce the results of Criado-Boado and colleagues using active inference. Because active inference is known as a good computational candidate to account for human behaviour, the main contribution of our simulation is to have provided the beginning of a behaviourally plausible explanation for the computation that may underwrite the results of Criado-Boado and colleagues. The explanation for the correlation we simulated is simple: the more complex the stimuli, the more exploration there is, and the more exploration there is, the more transitions are observed and therefore the more dispersion there is in the B2 parameters (i.e., the mappings are less deterministic). Cultural complexity thus has the consequence of “loosening” the learning of transitions among cultural motifs, and so renders learning more flexible (i.e., opens the agent to exploring novel shapes), which is a phenomenon discussed in relation to creativity (Van de Cruys and Wagemans, 2011; Veissière et al., 2020). There is more complexity and variety in the experienced transitions amongst hidden states, or more elaborate hierarchical structure, which in turn facilitates learning more complex and varied models of the world. In experiment 2, we simulated a simple visual classification task in which we reused the C-PAST trained in experiment 1. Here again, increased flexibility in learning prove useful. We measured success rate (hits non-hits) in a simple visual discrimination task under each C-PAST. The overall observation was that C-PASTs acquired during the exploration of more complex artefacts lead to better performance in the discrimination task.

Crucially, our results on the relation between cultural complexity and the “loosening” of the learning of transitions among cultural motifs are consistent with archaeological observations. For instance, in neolithic contexts, it has been observed that relatively uniform ceramic decorations increase the diversity of the decoration over time. For example, we can structurally identify Neolithic societies in Central Europe for which oldest phase uniform decorations are in use over a large area. In the following phase, this uniformity dissolves, which correlates with increase in decorations variability. In the case of Linear Pottery, this is associated with spin-offs of individual farmsteads from the central settlement around 5100 B.C. and increased generational independence (Shennan and Wilkinson, 2001). A similar phenomenon can be seen for the large-scale Globular Amphora phenomenon with a broadening of ritual activities around 3,000 BC (Müller, 1996). From 2500 B.C. onwards, cyclical increases and decreases in motif variation are observed for the Bell Beakers, which can be linked to an intentional renewed restriction of cultural diversity usually occurring every 150 years or so. Since comparable changes in diversity are also probable in the Bronze Age (cp. Staniuk, 2020), we should be able to identify a fundamental phenomenon for illiterate societies. The learning changes observed in the simulations offer at least one of several explanatory patterns for the archaeological observations described in the example.

The purpose of experiment 1 and 2 was to demonstrate the feasibility of an active inference based archaeological study of the effect of material culture on cognition. In future work, we plan on using the computational paradigm developed here to test empirically the correlations observed in our simulated experiment. Even though we used very simple tasks in experiments 1 and 2 for illustrative purposes, nothing prevents us from designing more complex simulation scenarios that can be used to model participant's performance in richer environments. Indeed, the likelihood remapping strategy we employed in this paper, because it builds environments based on a single type of hidden states, makes it possible to design complex 2D or 3D training environments and to transfer the learning of the model across different such environments.

Projected iterations of this new experimental paradigm could address at least four important and interlocking issues. The first, and most obvious, is to explore the effects of different material structures and practises on learning and attention. This could be done with contemporary artefacts characteristic of different culture, which we could use to test cross-cultural variations in visual attention styles. Such future studies should be informed by studies on cultural differences in physical objects perception (e.g., Masuda and Nisbett, 2001, 2006; Kitayama et al., 2003; Ishii et al., 2014). The second is to explore learning and transmission in whole populations of active inference agents. The third is to look at how learning that is achieved in one such generation and passed on to another influences the design of the environment itself—the so-called “trans-generational bottleneck” whose importance in the domain of language change has been the subject of much recent experimentation (for a review, see Smith and Kirby, 2008). Here, there is an opportunity to confront the real historical record with predictions made on the basis of the simulations. The fourth—and potentially the most revealing—would be to explore the principal dimensions along which variations in material culture and patterned practises impact learning and attention, using this to drive new (more functionally revealing) ways of grouping and taxonomising the real socio-historical record. For example, we predict that important variations will flow from the way different material designs manipulate sensory surprise at different levels of abstraction and processing.

Summing up, we have described a new experimental pipeline for exploring links between active inference and changing cultural complexity. These links are, we hypothesise, mediated by changing patterns of attention—patterns that can be trained and enforced by the structural and decorative complexity of the objects we encounter. In future work using this pipeline, we hope to discover more of the hidden variables and deep guiding principles linking material culture to changing patterns of thought and reason.

## Data Availability Statement

The original contributions presented in the study are included in the article/Supplementary Material, further inquiries can be directed to the corresponding author.

## Author Contributions

All authors contributed to the redaction of the manuscript. AT, BM and AC contributed to the development and writing of the code.

## Funding

Researchers on this article were supported by an Australian Laureate Fellowship project A Philosophy of Medicine for the 21st Century (Ref: FL170100160) and by a Social Sciences and Humanities Research Council doctoral fellowship (Ref: 752-2019-0065) (AC), by a PhD studentship from the Sackler Foundation and the School of Engineering and Informatics at the University of Sussex (AT); by an EPSRC PhD Studentship (BM), by a GAIN-Xunta de Galiza Groups of Excellence 2020 (FC-B), and by Horizon 2020 European Union ERC Advanced Grant XSPECT - DLV-692739 (AC). AT is grateful to the Mortimer and Theresa Sackler Foundation, which supports the Sackler Centre for Consciousness Science.

## Conflict of Interest

The authors declare that the research was conducted in the absence of any commercial or financial relationships that could be construed as a potential conflict of interest.

## Publisher's Note

All claims expressed in this article are solely those of the authors and do not necessarily represent those of their affiliated organizations, or those of the publisher, the editors and the reviewers. Any product that may be evaluated in this article, or claim that may be made by its manufacturer, is not guaranteed or endorsed by the publisher.
